# Development of Recombinant Antigen Array for Simultaneous Detection of Viral Antibodies

**DOI:** 10.1371/journal.pone.0073842

**Published:** 2013-09-13

**Authors:** Yi Liu, Fengling Yu, Haiyan Huang, Jinxiang Han

**Affiliations:** 1 Pediatric Research Institute, Qilu Children’s Hospital of Shandong University, Ji’nan, China; 2 Clinical Laboratory, Qilu Children’s Hospital of Shandong University, Ji’nan, China; 3 Key Laboratory for Biotech-Drugs of the Ministry of Health, Shandong Medicinal Biotechnology Center, Shandong Academy of Medical Sciences, Ji’nan, China; Weizmann Institute of Science, Israel

## Abstract

Protein microarrays have been developed to study antibody reactivity against a large number of antigens, demonstrating extensive perspective for clinical application. We developed a viral antigen array by spotting four recombinant antigens and synthetic peptide, including glycoprotein G of herpes simplex virus (HSV) type 1 and 2, phosphoprotein 150 of cytomegalovirus (CMV), Rubella virus (RV) core plus glycoprotein E1 and E2 as well as a E1 peptide with the optimal concentrations on activated glass slides to simultaneously detect IgG and IgM against HSV1, HSV2, CMV and RV in clinical specimens of sera and cerebrospinal fluids (CSFs). The positive reference sera were initially used to measure the sensitivity and specificity of the array with the optimal conditions. Then clinical specimens of 144 sera and 93 CSFs were tested for IgG and IgM antibodies directed against HSV1, HSV2, CMV and RV by the antigen array. Specificity of the antigen array for viral antibodies detection was satisfying compared to commercial ELISA kits but sensitivity of the array varied relying on quality and antigenic epitopes of the spotting antigens. In short, the recombinant antigen array has potential to simultaneous detect multiple viral antibodies using minute amount (3 µl) of samples, which holds the particularly advantage to detect viral antibodies in clinical CSFs being suspicious of neonatal meningitis and encephalitis.

## Introduction

Protein microarray, a high-throughput and powerful detection tool in medical research, is characterized methodologically as that of gene microarray, including robotic printing of probe targets, hybridization with samples of interest, and detection of interaction by high-resolution scanning and image analysis [1], [2]. Up to now, protein microarrays have been applied to analyze serum and other biological fluids in different contexts including protein-protein interaction, cancer profiling, autoimmune and infectious diseases [3]–[7].

Herpes simplex virus type 1 and 2 (HSV1 and HSV2), human cytomegalovirus (CMV), rubella virus (RV), are the important pathogens causing human vertically transmitted infections even neonatal meningitis and encephalitis [8]–[9]. Determination of the viral infection is currently depended on virus isolation, antigen measurement, nucleic acid detection and serological methods [10]–[12]. Enzyme-linked immunosorbent assays (ELISAs) are exceptionally sensitive and specific for detecting the concentration of proteins in complex biological fluids of blood, cerebrospinal fluid and others. ELISAs are routinely utilized to obtain serological proof of previous and current infection, with at least 100 µL of samples for single target protein so that large sample volumes are needed for serological diagnosis [6]. To overcome the constraints, protein microarrays have been developed to detect antibodies against infectious viruses by using crude extracts, semi-purified microbial antigens, and recombinant proteins with higher concordance rates between microarray assays and ELISAs [13]–[15]. To further explore the suitability of microarray platform for serodiagnostic purposes, we designed an antigen array immunoassay based on in-house recombinant proteins of HSV1, HSV2, CMV and synthetic peptides of RV. The main advantage is simultaneous determination of multi-antibodies from 3 µL of serum or cerebrospinal fluid (CSF).

## Materials and Methods

### Antigens Preparation

Specific recombinant proteins, HSV1 glycoprotein G (HSV1-gG), HSV2 glycoprotein G (HSV2-gG), and CMV phosphoprotein 150 (CMV-pp150) were expressed and purified as described previously [16]–[18] at Shandong Institute of Virology and Institute of Microbiology, Shandong Academy of Medical Sciences. Briefly, HSV1-gG (region from 52 aa –181 aa) was cloned and transferred to pBS221 expression vector from HSV type 1 stocker strain. HSV1-gG protein was induced in *E.coli* DH5α host strain by increasing temperature, and then was determined by SDS-PAGE and Western blot. The purity of recombinant HSV1-gG over 90% was obtained after purification through ion exchange [16]. HSV2-gG (region from 345 aa –584 aa) was constructed into pET-30a vector. The protein was expressed in BL21 *E.coli* through adding IPTG to 1 mmol/L and purified by his-tag purification and identified by Western blot [17]. Recombinant protein of human CMV phosphoprotein 150 fragment (CMV-pp150, region from 459 aa −691 aa) was constructed into pET-11a vector. The recombinant protein was induced with IPTG in BL21 *E.coli* host strain. After purification of DEAE-Sepharose FF Ion exchange and then Phenyl-Sepharose CL-4B, the CMV-pp150 protein was identified by SDS-PAGE and Western blot [18]. For comparison, commercial recombinant antigens of gG-1, gG-2 (expressed in *S.cerevisiae*) and pp150 expressed in *E.coli* were purchased from ViroState Inc (Portland, ME, USA).

Recombinant RV core+E1E2 protein (RV-E1E2) expressed in mammalian cells was purchased from ViroState Inc (Portland, ME, USA). An 18-amino-acid conserved peptide (RV-P, region from 248 aa −265 aa) from RV E1 antigen was designed (ATPERPRLRLVDADDPLL) after analyzing the antigenic epitopes of RV conserved E1 gene. Then it was synthesized at the Beijing Biosynthesis Biotechnology Company.

### Patient Samples and Reference Method

One hundred forty four human sera and 93 CSFs were provided by the Shandong Institute of Virology, which obtained from a heterogeneous group consisted of a majority of hospitalized patients and some outpatients, including 73 females 70 males of ages ranging from 1 month to 68 years from March, 2004 to December, 2005. All samples were determined serologically for IgG and IgM against HSV1, HSV2, CMV, RV by enzyme-linked immunosorbent assays (CanAg Diagnostics AB Majnabbe Teminal, Sweden) according to the manufacturer’s instructions.

### Ethics Statement

This work of retrospective human sera and CSFs for development of viral antigen array was approved by the Medical Ethics Committee of the Qilu Children’s Hospital of Shandong University and Shandong Academy of Medical Sciences. All patients or their parents gave their written informed consent before the examination was performed. The relevant regulations and institutional polices were followed strictly.

### Recombinant Antigen ELISA Assay

Checkerboard titration was initially performed to optimize the working concentrations of the coating antigens and sera. Recombinant antigens of HSV1-gG, HSV2-gG, CMV-pp150, and RV-E1E2, synthetic peptide RV-P diluted to optimal concentrations in 0.1 M carbonate-bicarbonate buffer (pH 9.6) were used to coat the 96-well flat bottom microliter plate (Nunc F96 Maxisorp, Denmark). The coated plates were incubated at 4°C overnight and then washed five times with washing buffer (0.01 mol/L PBS containing 0.05% Tween-20[v/v], pH 7.2). Blocking solution (0.01 mol/L PBS containing 0.05% Tween-20[v/v] and 1% BSA[w/v]) was used to block the unsaturated sites at 37°C for 1 h and subsequently washed five times with washing buffer. 1∶20 diluted positive sera were added to the wells and incubated at RT for 30 min following by washing 5 times. The plates were incubated with 1∶3000 diluted HRP conjugated goat anti-human IgG (Beijing ZSGB Bio Inc.) at 37°C for 30 min. After washing five times with washing buffer, the substrate solution (100 µg/ml of tetramethylbenzidine (TMB) (Sigma)) in citrate buffer (pH5.0), containing hydrogen peroxide (H_2_O_2_) 1 µl per 1 ml was added to the plates and incubated at 37°C for 15 min. The color reaction was stopped by adding 2 M sulphuric acid and then OD was determined at 450 nm on microplate reader.

### Preparation of Poly-L-lysine-coated Slides

Glass slides were immersed in 10% poly-L-lysine/PBS (v/v) and shaking at 60 rpm on a shaker for 1 hour, and then washed by ddH_2_O. The poly-L-lysine-coated slides were centrifuged to dry and stored for 2 weeks before using them.

### Fabrication of Recombinant Antigen Array

Viral antigens, human IgG or IgM were diluted using PBST (0.01 mol/L PBS with 0.01%Tween-20[v/v]) to different concentrations and spotted onto poly-L-lysine-coated slides by using a contact-printing robot (PixSys5500, Cartesian Inc., Newton, MA). Arrays consisted of a 4×5 matrix that including four viral antigens, human IgG (50 mg/ml, Sigma) or IgM(500 mg/ml, Sigma) as the positive control, and human recombinant in-house osteogenic protein-1 (OP-1) as the negative control, which were printed in four replicates. The transferred volume of each pin was 10 nL of antigens and the printed diameter of each spot was 120 µm. Printing was carried out in a cabinet at 20°C-25°C and 80% humidity. Printed slides were dried in the cabinet at room temperature and then stored at 4°C for use.

### Processing of Antigen Array

Antigen array slides were incubated in a solution of 1% bovine albumin (BSA) in PBST for 30 min at room temperature to block nonspecific antibody binding and then immersed into washing buffer (0.01 mol/L PBS containing 0.05% Tween-20) to wash for 3 times and 15 sec each time; then reacted with 3 µL sample of serum or CSF for 30 min at 37°C covered with a cover slide (Hybrislip hybrization cover slides, Invitrogen), following by second washing for 3 times 15 sec each time for and then incubated for 30 min at 37°C with 3 µL of 33 µg/ml Cy3-anti-human-IgG (Sigma) or 5 µg/ml Cy3-anti-human-IgM (Sigma) which diluted with blocking solution. The slides were washed and dried for scanning.

### Data Analysis

The fluorescence was scanned by using a fluorescence scanner (Scanarray 4000, General Scanning Inc, USA) at 90% laser intensity, 90% PMT for sera and 100% laser intensity, 100% PMT for CSFs. The QuantArray software (version 3000) was used to analyze the data. The differences in the antibodies relativities between antigen array and ELISA were analyzed using the Chi square test. The diagnosis sensitivity, specificity, positive predictive value and negative predictive value were calculated relative to the results of ELISA assays.

## Results

### Determination of Cy3 Labeled Secondary Antibodies Concentrations

To determine the optimal concentrations of Cy3-anti-human-IgG/IgM, purified human IgG and IgM were diluted and spotted onto poly-L-lysine-coated slides in 5 replicates, containing a positive control (0.1 mg/ml IgG or IgM) and a regent control (PBST). Fluorescence signal was detected after hybridization with different concentrations of Cy3-anti-human-IgG/IgM (1–100 µg/ml). The optimal concentration of Cy3-anti-human-IgG/IgM was 33 and 5 µg/ml, separately.

**Figure 1:**
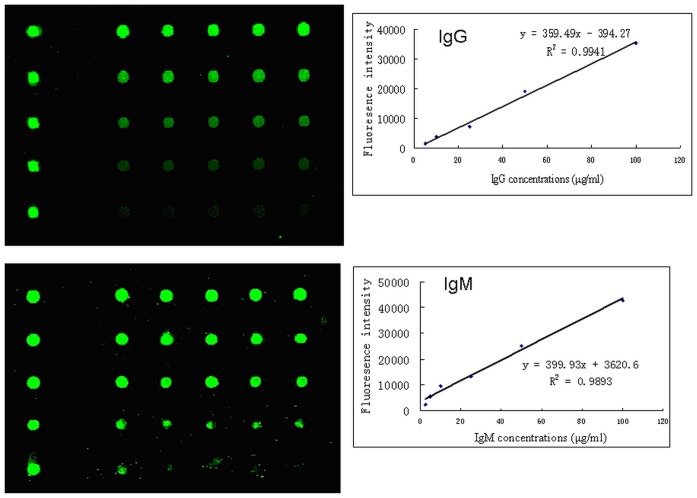
Dose response curves of fluorescence intensity and concentrations of human IgG and IgM. A. The fluorescence signals scanned at different concentrations of human IgG 5, 10, 25, 50, 100 µg/ml; B. The fluorescence signals scanned at different concentrations of human IgM 2.5, 5, 10, 25, 50,100 µg/ml. The equation and the coefficient of determination (R^2^) are indicated for each curve.

The nearly linear concentration curves were obtained in the range of 5 µg/ml to100 µg/ml for IgG and 2.5 µg/ml to100 µg/ml for IgM. The coefficients of determination (R^2^) were close to 1 (0.9941 for IgG and 0.9893 for IgM, separately). Fluorescence intensity was nearly saturation when concentrations of IgG and IgM were above 100 µg/ml (Fig. 1).

### Selection of Optimal Antigen Concentrations

To select the best concentrations of each antigen for spotting, a range of concentrations from 1 to 50 µg/ml of each antigen including HSV1-gG, HSV2-gG, CMV-pp150, mixture of RV-E1E2 and RV-P were selected to spot onto poly-L-lysine-coated slides in 5 replicates. Hybridization was performed with positive reference sera in ELISA kits. The optimal concentrations of Cy3-anti-human-IgG (33 µg/ml) and Cy3-anti-human-IgM (5 µg/ml)) were added to collect fluorescence signals. The in-house antigens showed comparable antigenicity but with lower fluorescent intensities than the commercial antigens (data not showed). Finally, the optimal concentrations of HSV1-gG, HSV2-gG, CMV-pp150 were 12.5, 25 and 12.5 µg/ml. For the optimal antigen of RV, the 100 µg/ml of RV-E1E2 and 10 µg/ml of RV-P (written as RV-EP) gave the best result.

**Figure 2:**
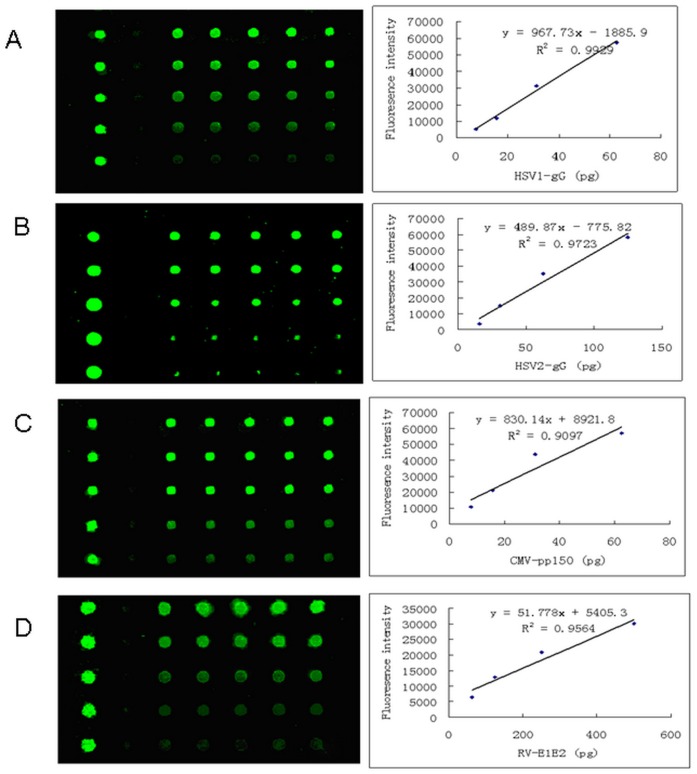
Calibration curves of fluorescence intensity and concentrations of four recombinant viral antigens. A. The fluorescence signals scanned at different concentrations of antigen HSV1-gG from 1.56, 3.125, 6.25, 12.5, 25 µg/ml to 50 µg/ml; B. The fluorescence signals scanned at different concentrations of antigen HSV2-gG from 1.56, 3.125, 6.25, 12.5, 25 µg/ml to 50 µg/ml; C. The fluorescence signals scanned at different concentrations of antigen CMV-pp150 from 1.56, 3.125, 6.25, 12.5, 25 µg/ml to 50 µg/ml; D. The fluorescence signals scanned at different concentrations of antigen RV-EP from 0.31, 0.625, 1.25 2.5, 5, 10 µg/ml; the equation and the coefficient of determination (R^2^) are indicated for each curve.

The minimum of four antigens to detect IgG and IgM were 31 pg for HSV1-gG and HSV2-gG, 15 pg for CMV-pp150 and 31 pg for RV-EP, respectively. (Fig. 2).

### Specificity of the Recombinant Antigen Array

**Figure 3:**
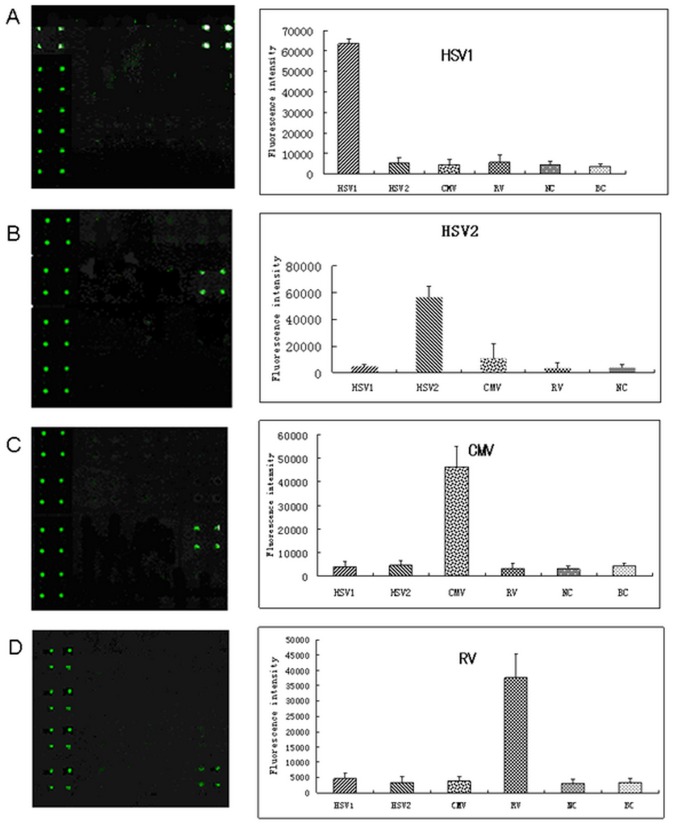
Specificity of the recombinant antigen array. A. B. C. D. Fluorescence signals scanned and analysis results after hybridization with IgG positive reference sera against HSV1 (A), HSV2 (B), CMV (C), RV (D), separately.

Four antigens of HSV1-gG, HSV2-gG, CMV-pp150 and RV-EP were spotted on poly-L-lysine-coated slides in 4 replicates to fabricate the antigen array, and the specificity of the array was evaluated by using the positive reference sera in ELISA kits, containing different antibodies against HSV1, HSV2, CMV and RV. It showed no cross reaction with any non-specific probes on the microarray (Fig. 3).

### Repeatability of the Recombinant Antigen Array

**Figure 4:**
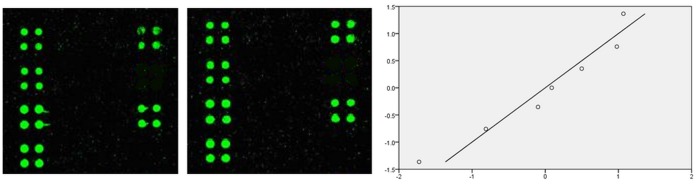
Repeatability of the recombinant antigen array. Fluorescence images and Q-Q plot analysis after hybridization with the positive serum of HSV1 and CMV on two different batches of array slides.

The repeatability of the antigen array was evaluated by using different batches of array slides and one serum with positive IgG reactivities for HSV1 and CMV, showing comparable and satisfying results (Fig. 4).

### Antigen Array for Detection of Viral IgG

**Figure 5:**
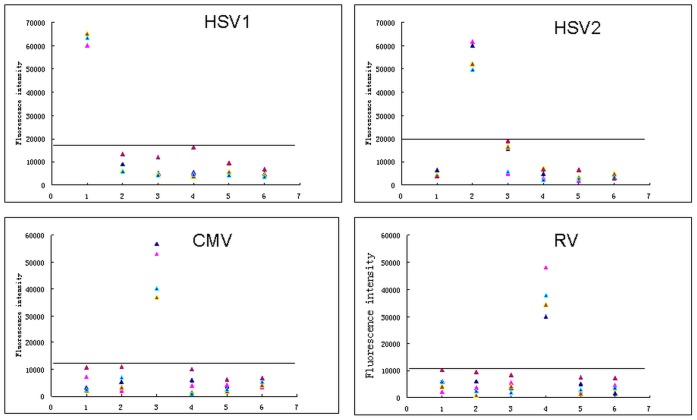
Determination of the cutoff value for IgG reactivity against HSV1, HSV2, CMV and RV. Scatter plots of IgG reactivities for different specific antigens of HSV1-gG, HSV2-gG, CMV-pp150, RV-EP compared to other unspecific antigens and blank control when positive reference sera of HSV1, HSV2, CMV and RV were used.

We detected the IgG reactivities of 144 sera and 93 CSFs against HSV1, HSV2, CMV, and RV by using the antigen array and compared the results with ELISA assays. The sensitivity, specificity, positive predictive value and negative predictive value of IgG for each antigen were calculated. The cutoff values of the array for each antigen were determined separately using the negative and positive reference sera in ELISA kits. Shortly, the mean fluorescent intensities of the negative and positive reference sera bound to each antigen were measured. The positive reference sera indicated much higher fluorescent signals than that of the negative sera. Finally, the fluorescent intensities of the negative reference sera bound to each antigen (background values) plus 3 standard deviations were regarded as cut-off values of each antigen. The reactivity of specimens was classified as either positive or negative according to the presence of IgG concentrations above or below the cutoff value (Fig. 5).

**Figure 6:**
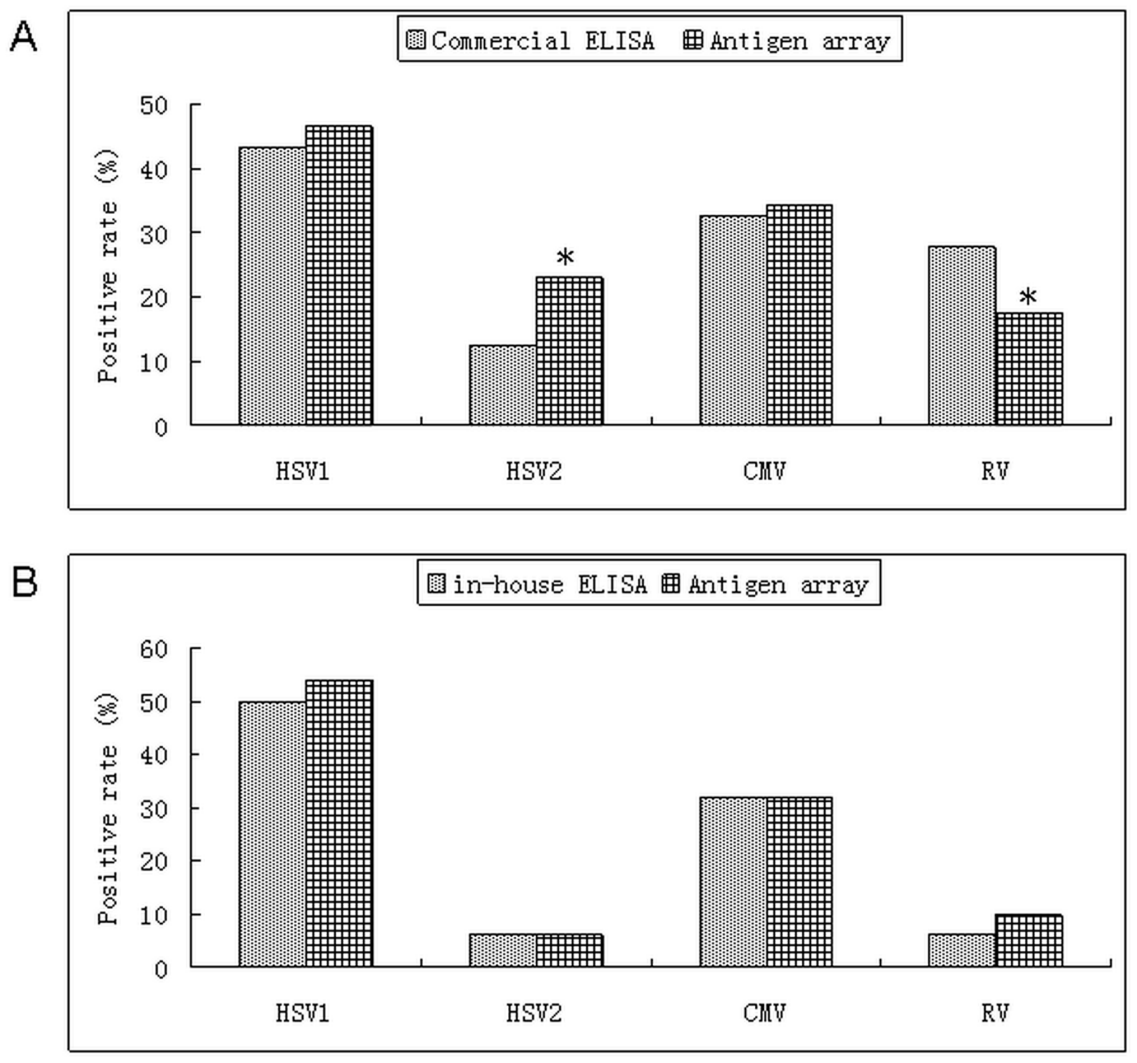
Comparison of ELISAs and antigen array for serum IgG reactivity. A. Comparison of commercial ELISA and antigen array; B. Comparison of in-house ELISA and antigen array *indicated P<0.05.

The IgG positive reactivity against HSV1, HSV2, CMV and RV in sera was 46.43, 22.92, 34.23, and 17.36% with antigen array; while 43.06%, 12.5%, 32.64% and 27.78% with commercial ELISA assays, separately. There were no statistic difference between the two assays (P = 0.7281 for detection of HSV1 IgG and P = 0.8026 for detection of CMV IgG) in sera. But for detection of HSV2 and RV, P values were 0.0206 and 0.0345 with two assays. The diagnostic sensitivity, specificity, positive predictive value and negative predictive value were calculated with ELISAs as gold standards (Fig. 6A, Table1).

**Table 1:** Evaluation of antigen array assay for serum IgG reactivity.

Virus	HSV1	HSV2	CMV	RV
Sensitivity (%)	85.48	38.89	82.98	20.00
Specificity (%)	85.37	79.37	89.69	83.65
Positive predictive value(%)	81.54	21.21	80	32.00
Negative predictive value(%)	88.61	90.90	92.54	73.11

The two methods were then tried to detect viral IgG in CSFs and the results were showed in Table 2. P value was calculated for HSV1 and CMV IgG but there was no assessment for HSV2 and RV due to zero positive specimens (Table 2).

**Table 2:** Comparison of ELISA and antigen array for CSF IgG reactivity.

Virus		HSV1	HSV2	CMV	RV
ELISA(IgG)	Positive(%)	5 (5.38)	0 (0)	3 (3.23)	0 (0)
	Negative(%)	88 (94.62)	93 (100)	90 (96.77)	93 (100)
Antigen array(IgG)	Positive(%)	3 (3.23)	1 (1.08)	3 (3.23)	0 (0)
	Negative(%)	90 (96.77)	92 (99.92)	90 (96.77)	93 (100)
*P*		0.7178	n	0.6781	n
Sensitivity (%)		60.00	n	66.67	n
Specificity (%)		100.00	n	98.89	n

n. not calculated.

### Antigen Array for Detection of Viral IgM

The viral IgM relativities against the four viruses were detected from 144 sera and 93 CSFs by using two methods, separately. The positive and negative IgM relativities were determined following the same way as that of IgG detection. The positive rates for HSV1 and CMV were comparable and there was no significance between the two methods. The diagnostic sensitivity and specificity were calculated for HSV1 and CMV with ELISAs as gold standards. But the analysis of sensitivity and specificity was not carried out for HSV2 and RV due to insufficient number of positive specimens (Table 3).

**Table 3:** Comparison of ELISA and antigen array for serum IgM reactivity.

Virus		HSV1	HSV2	CMV	RV
ELISA(IgM)	Positive(%)	6 (4.17)	2 (1.39)	16 (11.11)	5 (3.47)
	Negative(%)	138 (95.83)	142 (98.61)	128 (88.89)	139 (96.53))
Antigen array(IgM)	Positive(%)	3 (2.08)	0 (0)	10 (6.94)	0 (0)
	Negative(%)	141 (97.92)	144 (100)	134 (93.06)	144 (100)
*P*		0.5116	n	0.5255	n
Sensitivity (%)		50.00	n	80.00	n
Specificity (%)		100.00	n	90.36	n

n. not calculated.

Considering the significant differences of HSV2-IgG and RV-IgG detection between the antigen array and ELISA kits, we used the identical antigens of HSV1-IgG, HSV2-IgG, CMV-pp150 and RV-EP to coat the plates as in-house ELISA assays to compare two methods with 50 clinical sera. Three of them shared positive IgG antibodies against HSV1 and CMV. The high consistencies were observed and P values were 0.6889, 0.6737,1.0 and 0.7492 for HSV1, HSV2, CMV and RV (Fig. 6B).

## Discussion

We established an antigen microarray based on ELISA assay and took ELISA as a gold standard for comparison of two methods in this study. The ELISA kits used were made from whole virus lysate. To be consistent with the condition of ELISA assay, the crude extracts of viruses were initially chosen to set up the array, but the striking cross-reactivities were observed when sera were hybridized with the antigens on the array (data not showed). Ideal protein microarrays should apply highly-purified antigens [19]. Since purified recombinant antigens have indicated good specificity and antigenicity in immunoassay [20]–[22], moreover antigens expressed in *Escherichia coli* also showed satisfying specificity [21]–[23], we designed the antigen array based on recombinant viral specific antigens expressed in *E.coli* and evaluated its preliminary application for detection of IgG and IgM directed against various viral antigens, including HSV1, HSV2, CMV and RV.

For HSV1 and HSV2, the glycoprotein G has been applied to develop new methods for discrimination between HSV1 and HSV2 antibodies in clinical application [24]. CMV UL32 (encoding pp150) has been reported as major target antigens for immunoassay [25]. We cloned and expressed HSV1-IgG, HSV2-IgG, and CMV-pp150 proteins in *E.coli*, separately. After purification, over 90% pure proteins were obtained and utilized to fabricate this antigen array. The antigenicity and specificity of in-house recombinant antigens were initially compared with commercial recombinant antigens.

Recombinant RV antigen of core+E1E2 purchased from the commercial company was demonstrated the lower detection sensitivity of IgG and IgM against RV in our pre-experiment. RV E1 glycoprotein was found not only to be immunodominant, but also the only antigen recognized by all the antibody isotypes [26], [27]. To increase the detection sensitivity, an 18-amino-acid conserved polypeptide containing 1–2 strong antigenic epitopes of RV E1 antigen (RV-P) was designed and synthesized. Its antigenicity was superior to the recombinant RV-E1E2 in our pre-experiment (data not show). Considering the small polypeptide molecular might be influenced by steric hindrances of other recombinant antigens, the recombinant antigen and E1 polypeptide were mixed to be the printing antigen of RV. The comparable results were obtained.

In this study, the dose-response IgG and IgM calibration curves were firstly measured, and then extended to the recombinant viral antigens to determine their optimal spotting concentration, spotting buffer, secondary antibodies, and so on. The linear trend lines were showed and the correlation coefficients were close to 1. The minimum of four antigens to detect IgG and IgM were 31 pg for HSV1-gG and HSV2-gG, 15 pg for CMV-pp150 and 31 pg for RV-EP, respectively. The ELISA based-array allows to simultaneous detect multiple viral antibodies using minute amount (3 µL) of samples, which holds the particularly advantage to detect viral antibodies in clinical CSFs being suspicious of neonatal meningitis and encephalitis.

Our results showed that the recombinant antigen array can be used to detect the presence and absence of viral antibodies in human sera and CSFs. When this antigen array was used to detect HSV1-IgG and IgM, CMV-IgG and IgM, there was no statistical significance between array and commercial ELISA kits. The sensitivity of the antigen array for detection of HSV1 and CMV IgG was 85.48% and 82.98%; for HSV1 and CMV IgM was 50% and 80% with inadequate positive samples. When HSV2 and RV antibodies were detected, the sensitivities were only 38.89% and 20%. Considering the commercial ELISA kits were made from native whole antigens, we inferred that the inconsistent results were mainly from the different coating antigens. In this case, the in-house ELISA assays was set up and compared with the results from the array using 50 of those sera, the very consistent results were obtained, highlighting further the importance of coating antigens’ quality and consistence for antigen array and ELISA assay.

This array was also tried to evaluate the presence and absence of viral IgM antibody in sera and CSFs, demonstrating its suitability for IgM detection. But the number of positive samples available was too small to draw conclusions regarding to the clinical performance. Our study provides alternative selection of in-house antigens to ELISA assays. More importantly,the array is flexible and easy to enlarge and modified for clinical requirements.
